# Plasma neurofilament light chain as a potential biomarker of neurodegeneration in murine brain

**DOI:** 10.1093/toxres/tfad063

**Published:** 2023-08-01

**Authors:** Tomoya Sano, Yasushi Masuda, Hironobu Yasuno, Tadahiro Shinozawa, Takeshi Watanabe

**Affiliations:** Drug Safety Research and Evaluation, Takeda Pharmaceutical Company Limited, 26-1 Muraoka-Higashi 2-Chome, Fujisawa, Kanagawa 251-8555, Japan; Drug Metabolism and Pharmacokinetics Research Laboratories, Takeda Pharmaceutical Company Limited, 26-1 Muraoka-Higashi 2-Chome, Fujisawa, Kanagawa 251-8555, Japan; Drug Safety Research and Evaluation, Takeda Pharmaceutical Company Limited, 26-1 Muraoka-Higashi 2-Chome, Fujisawa, Kanagawa 251-8555, Japan; Drug Safety Research and Evaluation, Takeda Pharmaceutical Company Limited, 26-1 Muraoka-Higashi 2-Chome, Fujisawa, Kanagawa 251-8555, Japan; Drug Safety Research and Evaluation, Takeda Pharmaceutical Company Limited, 26-1 Muraoka-Higashi 2-Chome, Fujisawa, Kanagawa 251-8555, Japan

**Keywords:** neurofilament light chain, neurotoxicity, trimethyltin, mice

## Abstract

Reliable fluid biomarkers for evaluating neurotoxicity have yet to be established. However, recent studies have reported neurofilament light chain as a fluid biomarker of several neurodegenerative disorders. In this study, we investigated changes in the cerebrospinal fluid and plasma levels of neurofilament light chain in mice treated with trimethyltin as a neurotoxicant. Trimethyltin diluted with saline was administered by intraperitoneal injection to mice at dose levels of 0 (vehicle control), 1.0, and 2.6 mg/kg body weight (dosage volume: 10 mL/kg). At 3 or 7 days after administration, animals were euthanized by exsanguination under 2–3% isoflurane inhalation anesthesia. Increased neurofilament light chain levels in both the cerebrospinal fluid and plasma were observed in animals from the trimethyltin 2.6 mg/kg body weight group, which indicated the brain lesions including neuronal cell death. Animals from the trimethyltin 1.0 mg/kg body weight group exhibited changes neither in neurofilament light chain levels in the cerebrospinal fluid and plasma nor in the histopathology of the brain at any time point. These data indicate that plasma neurofilament light chain can serve as a useful peripheral biomarker for detecting brain lesions such as neuronal necrosis in mice.

## Introduction

Neurotoxicity has a major impact on drug development and is typically evaluated using multiple parameters.^1^ However, the lack of standardized and universally accepted fluid biomarkers makes the analysis of neurotoxicity quite challenging. Therefore, the development of novel translatable biomarkers of neurotoxicity is highly desired, not only to guide nonclinical drug development but also to ensure patient safety in clinical trials. Recent studies have reported the measurement of neurofilament light chain (NfL), a structural scaffolding protein of the axonal cytoskeleton of neurons in the nervous system,^2^ in the cerebrospinal fluid (CSF) and/or blood as a method of detecting neurotoxicity in rats, monkeys, and humans by using an ultrasensitive and reliable immunoassay based on single-molecule array technology (Simoa).^3–7^ In our previous rat study, chemically induced neuronal necrosis in the brain was successfully detected by increased serum NfL levels, which were correlated to the CSF NfL levels and brain histopathology.^5^ Thus, NfL is thought to be released from the damaged nerve tissues to extracellular space and subsequently into CSF, and then into the blood after the occurrence of neurotoxicity in the rat brain. NfL has also been used as a biomarker for assessing disease severity and progression or for monitoring therapy response in several neurological disorders in mice and human studies.^8–10^ However, there are no reports to confirm the alteration of NfL levels in mice treated with neurotoxicants. It is therefore crucial to demonstrate the usability of NfL in nonclinical safety evaluation using mice as well as other animal species and to propose a suitable tool for neurotoxicity assessment.

In the present study, trimethyltin (TMT), an organotin compound with neurotoxic effects in the hippocampus (Hipp), was selected to obtain the central nervous system (CNS) insult in mice. The underlying mechanisms of TMT-induced hippocampal neurodegeneration include oxidative stress, altered calcium homeostasis, neuronal apoptosis, and inflammation.^11^ These molecular mechanisms of TMT-induced neuronal damage have been evaluated in vitro in mice, rats, and human cell lines,^11^ and TMT-induced neurodegeneration in mice after intraperitoneal administration is well characterized in the existing literature.^12^^,^^13^ Subsequently, we employed the Simoa assay to measure NfL levels in CSF and plasma and evaluated whether NfL can be utilized for detecting CNS toxicity, and especially microscopic neuronal necrosis in the brain of mice treated with TMT.

## Materials and methods

### Animals and treatments

This study was approved by the Institutional Animal Care and Use Committee (Approved study No. AU-00030827). We used 8-week-old male C57BL/6 J mice (The Jaxon Laboratory Japan, Inc.) with weights ranging from 21.5 to 25.4 g. The mice were selected using standardized normal values calculated from the body weights. These animals were allocated to 3 groups (control, low dose of TMT [TMT-L], and high dose of TMT [TMT-H]), each comprising 6 males necropsied at 3 (day 4) or 7 day (day 8) after a single intraperitoneal dose of TMT (TMT Chloride, Purity >98%, Tokyo Chemical Industry Co., Ltd, Tokyo, Japan). TMT Chloride diluted with saline at dose levels of 1.0 and 2.6 mg/kg body weight (TMT-L and TMT-H groups, dilution factor: 1.22, dosage volume: 10 mL/kg). The control group was given saline alone in the same manner. The TMT-H, which produces overt neuronal necrosis in the brain, was selected based on doses reported in the literature.^12–14^ Additionally, the TMT-L was also selected as a potential nontoxic dose.^13^ The animals were group-housed in polycarbonate cages equipped with chew toys (I chew, ASAP and Nylon Bone, Bio Serv., NJ, USA) as animal enrichment devices. The conditions of the room were as follows: temperature of control range: 20–26°C; relative humidity of control range: 40–70%; air exchange: 10–25 times per hour; and a 12-h light/dark cycle. The animals were allowed free access to a pelleted laboratory animal diet (ce-2, CLEA Japan, Inc., Tokyo, Japan) and tap water. Clinical signs and body weights were checked once daily.

### Collection of CSF, blood, and the brain samples

Before the collection of brain tissues, blood, and CSF samples at each necropsied time point, the animals were anesthetized with 2–3% isoflurane in oxygen as a carrier gas. Blood samples (0.4 mL) were collected from the abdominal aorta using a syringe with anticoagulant (3% [v/v] .1 M EDTA-2Na) under isoflurane anesthesia prior to necropsy. The blood samples were subsequently centrifuged at 18,000 × *g* for 1 min at 4°C to obtain plasma. Immediately after euthanasia by exsanguination, the skin and muscles around the back of the neck were removed and the dura mater of the cisterna magna was exposed. CSF samples were collected via cisterna magna puncture using a syringe and needle. Then, the animals were necropsied and the collected whole brain was weighed and fixed in 10% neutral buffered formalin for subsequent microscopic examination.

### Histopathological examination

The brain was trimmed for 5-level sections (Levels 1–4 and 6) in accordance with the STP position paper,^15^ embedded in paraffin, and sectioned. The 4-μm-thick sections were stained with hematoxylin and eosin (H&E). Staining with Fluoro-Jade C (FJ-C), a neuronal cell death marker, was additionally performed for the brain sections of all animals in the TMT-H group.

### Measurement of NfL levels in CSF and plasma

NfL levels were measured using the commercially available Simoa NF-light Advantage Kit (# 103400, Quanterix Corporation, MA, USA) on the Simoa SR-X Biomarker Detection System (Quanterix Corporation, MA, USA). To each well of the 96-well plate, 100 μL of sample/calibrator, 20 μL of biotinylated antibody detector, and 25 μL of antibody-coated paramagnetic beads were added. The plate was then incubated at 30°C with shaking at 800 rpm for 30 min on a Simoa Microplate Shaker (Quanterix Corporation, MA, USA). After the incubation, the beads in the wells were washed using the Simoa Microplate Washer (Quanterix Corporation, MA, USA) to retain the beads by a magnet during washing. After washing, 100 μL of streptavidin-β-galactosidase solution was added to the beads and incubated at 30°C with shaking at 800 rpm for 10 min. The well was washed again and 100 μL of Buffer B of the kit was added to each well. The plate was then incubated at 30°C with shaking at 800 rpm for 1 min. The beads were washed and the supernatant was aspirated by the plate washer. After the beads were allowed to dry for 10 min, the plate was then transferred to the Simoa SR-X analyzer. On the Simoa SR-X analyzer, automatically, the beads were resuspended in resorufin-β-D-galactopyranoside solution for signal generation, loaded onto the array, and the signals from bound analyte targets on the beads were analyzed to yield units of average enzymes per bead (AEB) as previously described.^16^ AEB values were analyzed by the software in the SR-X analyzer for quantification. Plasma and CSF samples were diluted 20-fold and 80-fold with Sample Diluent of the kit, respectively, and measured in duplicate. The calibrators were measured in triplicate in each run. The accuracy of lower limit of quantification ranged from 100 to 110% and the accuracy of upper limit of quantification ranged from 98 to 105%. The accuracy of other 5 calibrators was from 88 to 121%. Quality control (QC) samples in 2 levels provided with a kit were analyzed in duplicate in each run. The mean accuracy obtained with high-QC and low-QC samples was 84 and 100%, respectively. The inter-assay coefficient variation (CV) obtained with high-QC and low-QC samples of the kit was 2.8 and 7.6%, respectively. A control plasma sample was analyzed in triplicate in each run, demonstrating mean intra-assay CV of 5.5% and mean inter-assay CV of 11%.

### Statistical analysis

The data on NfL levels were tested by Williams’s test assuming a dose-related trend. The Williams’ test was conducted at the 2-tailed significance levels of 0.025. Analysis was performed using EXSUS (EPS Corporation, Tokyo, Japan). Correlations between CSF and serum NfL levels were determined using Pearson’s correlation coefficient.

## Results

### Clinical signs, body weight, and brain weight

In the TMT-H group, the body weight and brain weight were slightly decreased on days 4 and 8 (<10% difference from the mean values of the control group). The clinical signs for all mice from the TMT-H group included convulsions at day 2, and 3 animals showed recovery within a few days (Table 1). On day 8, convulsion had disappeared in the remaining animals from the TMT-H group. In the TMT-L group, there were neither body weight or brain weight changes nor abnormal clinical signs during the study period.

**Table 1:** Onset of convulsion, distribution, and severity of brain lesions and NfL data in the animals from TMT-H group.

			Convulsion	Distribution of neuronal necrosis^a^				Chromatolysis	Vacuolation	NfL (pg/mL)	
Test article	Animal No.	Necropsy timing	Timing	Hipp/DG	FrPT	Ent	Pir	Me5	STT	CSF	Plasma
Trimethyltin 2.6 mg/kg	1	Day 4	Days 2–4	±	−	−	−	−	−	5,482	2,804
	2	Day 4	Days 2–4	+	±	±	±	−	±	16,386	3,440
	3	Day 4	Days 2–4	±	±	±	±	±	±	8,120	4,898
	4	Day 4	Days 2–4	+	±	±	±	±	±	21,876	3,265
	5	Day 4	Days 2–4	+	±	±	±	±	±	17,519	5,600
	6	Day 4	Days 2–4	±	−	−	−	±	±	2,359	842
	7	Day 8	Days 2–4	±	−	−	−	−	±	63,279	3,736
	8	Day 8	Days 2–4, 6, and 7	±	−	−	−	−	−	19,179	1,429
	9	Day 8	Days 2–7	+	±	±	−	−	−	55,309	5,747
	10	Day 8	Days 2–7	±	±	−	±	−	±	23,494	3,472
	11	Day 8	Days 2–4	−	−	−	−	−	−	51	13
	12	Day 8	Days 2 and 3	−	−	−	−	−	−	412	19

−: no change or not remarkable finding, ±: minimal, +: mild, ++: moderate.

FrPT, frontopaietal cortex; Pir, piriform cortex; Me5, mesencephalic trigeminal nucleus.

^a^Distribution of neuronal necrosis was evaluated by H&E and FJ-C-stained slides.

### Histopathological examination

**Fig. 1:**
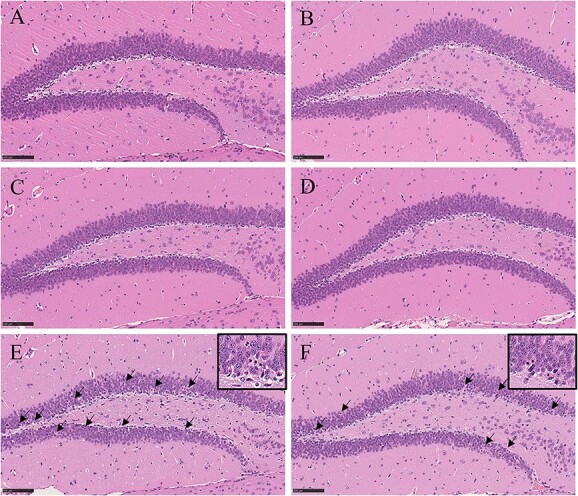
Representative photographs of the DG (H&E stain). There were no remarkable changes in the control group on days 4 a) and 8 b) and TMT-L group on days 4 c) and 8 d). Neuronal necrosis of DG was presented in the TMT-H groups at days 4 e) and 8 f) (bars: 50 μm). Arrows indicate necrotic neurons of the DG.

Individual histopathological findings in the brains of animals in the TMT-H group are shown in Table 1 and representative lesions are shown in Fig. 1.

In the TMT-H group, all animals showed neuronal necrosis in the Hipp, dentate gyrus (DG), frontoparietal cortex, entorhinal cortex (Ent), and/or pyriform cortex in the cerebrum at day 4 (Fig. 1e). FJ-C positive neurons were consistent with neuronal necrosis in the H&E staining sections. Additionally, chromatolysis of mesencephalic nucleus and/or vacuolation in spinal trigeminal tract (STT) were noted in almost all animals. On day 8, neuronal necrosis in the brain was observed in 4 out of 6 animals (Fig. 1e), but its distribution was in a more limited area when compared with that observed on day 4 (Table 1). Vacuolation of STT was infrequent, and chromatolysis of mesencephalic nucleus was not noted at this time point. The remaining 2 animals from the TMT-H group at day 8 did not show any lesions in the brain (Table 1).

**Table 2:** The levels of NfL in CSF and plasma following TMT administration.

	CSF (pg/mL)		Plasma (pg/mL)	
Test article	Day 4	Day 8	Day 4	Day 8
Control	389 ± 618	873 ± 1314	33.6 ± 18.7	24.2 ± 8.4
TMT-L	790 ± 954	959 ± 1818	20.1 ± 10.5	27.1 ± 10.8
TMT-H	11,957 ± 7,717^*^	26,954 ± 26,923^*^	3,475 ± 1,671^*^	2,403 ± 2,300^*^

^^*^^ *P* < 0.025 vs control.

No histopathological findings were observed in any of the brain tissues in the control and TMT-L groups (Fig. 1a–d).

### NfL levels in the CSF and plasma

The NfL levels in the CSF and plasma of the TMT-treated animals are shown in Tables 1 and 2.

In the TMT-H group, the apparent and statistically significant elevation of NfL levels in the CSF and plasma was confirmed on day 4 (~31-fold and 104-fold increase in the CSF and plasma, respectively) and day 8 (~31-fold and 99-fold increase in the CSF and plasma, respectively). Both CSF and plasma NfL levels were dramatically increased in all animals showing brain lesions, whereas there were no changes in NfL levels in 2 animals without brain lesions on day 8. However, individual levels of CSF and serum NfLs did not correlate in the TMT-H group (*r* = 0.579). In the TMT-L group, no significant differences in NfL levels in the CSF and plasma were observed.

## Discussion

We demonstrated that plasma NfL levels could be a marker for neurodegeneration in the brain in TMT-treated mice.

In the present study, all mice from the TMT-H group exhibited convulsions on day 2 and then recovered within a few days. The distribution and severity of the brain lesions including neuronal necrosis in this group decreased from days 4 to 8. It was reported that the initiation of seizure and the peak of Fluoro-Jade-positive neurons in the DG of mice occurred 2 days after TMT treatment (equivalent to day 3 in this study).^14^ Additionally, it is also known that TMT-induced neuronal loss of hippocampal DG in mice recover through the replacement of newly formed neurons by 14 days posttreatment.^17^ These results are consistent with the data obtained in this study.

Both CSF and plasma NfL levels in the TMT-H group were consistently increased in animals with histopathological lesions in the brain. In contrast, there were no changes in CSF and plasma NfL levels in the animals from the TMT-L group, which showed no abnormalities in clinical sings and brain histopathology. Thus, CSF and plasma NfL could be a reliable marker of brain lesions in the mouse, and blood NfL has a promising potential for less invasive detection of neurotoxicity in nonclinical studies. These data were also consistent with previous studies, which demonstrated the elevation of serum NfL levels in rats treated with various neurotoxicants^5^ and plasma NfL levels in murine models of neurodegenerative disease.^10^ Furthermore, blood NfL levels were reported to be strongly associated with the adeno-associated virus-induced dorsal root ganglion toxicity in rats and monkeys.^3^^,^^4^ The low variability of the baseline level in serum/plasma NfL was confirmed in this study, as well as in the previous studies in rats and monkeys,^4^^,^^5^^,^^18^ and contributed to the sensitivity of NfL in detecting neurotoxicity. Neurofilaments are released into the extracellular space when the brain tissue is damaged and subsequently into the CSF, which then leaks into the blood. CSF and plasma NfL levels were extremely high in affected mice in this study, both were not well correlated. A possible reason for the lack of correlation between CSF and plasma NfLs could be saturation of blood NfL because of rapid leakage a large amount of NfL from CSF to blood in this study.

Even though brain lesions showed a tendency of recovery on day 8, both CSF and plasma NfL levels in the animals with neurodegeneration were similar to those observed on day 4. This result indicates the persistence of NfL levels for several days after the protein’s release into the CSF and plasma. Therefore, it is assumed that brain lesions including neuronal necrosis did not occur throughout the study in 2 TMT-H group animals showing neither Fluoro-Jade-positive neurons nor NfL changes, although convulsion was observed like in the other animals from the TMT-H group. Given that changes in NfL levels were noted in the animals (Animal Nos 1 and 8) in which minimal neuronal necrosis was noted only in the Hipp and DG on day 4 or 8, plasma NfL can be considered a robust nonclinical marker for detecting acute neuronal cell necrosis of mouse brain with high accuracy. However, the translational value of NfL still needs to be examined because the data for evaluating neurotoxicity in the clinical setting are still limited. Recently, the Innovative Medicines Initiative Translational Safety Biomarker Pipeline (TransBioLine) project initiated the qualification of serum biomarkers including NfL to aid in the detection of acute drug-induced CNS toxicity in clinical trials (https://www.fda.gov/media/140339/download). The outcomes of these studies will play a major role in determining how to develop less neurotoxic medicines using novel biomarkers.

## Conclusions

In conclusion, plasma NfL is a useful biomarker for detecting neuronal cell death in the mouse brain with a sensitivity similar to that of histopathological examination. Considering the strong responses of the CSF and plasma NfL levels in mice, NfL will be a promising and useful blood-based biomarker for the evaluation of brain lesions including neuronal necrosis. Utilization of NfL in nonclinical safety assessment will facilitate the detection of neurotoxicity risk.

## Data Availability

All the data on the conclusion of this article are presented in the manuscript.

## References

[1] Walker AL , Imam SZ, Roberts RA. Drug discovery and development: biomarkers of neurotoxicity and neurodegeneration. Exp Biol Med. 2018:243(13):1037–1045. 10.1177/1535370218801309 PMC6434454 30253665

[2] Khalil M , Teunissen CE, Otto M, Piehl F, Sormani MP, Gattringer T, Barro C, Kappos L, Comabella M, Fazekas F, et al. Neurofilaments as biomarkers in neurological disorders. Nat Rev Neurol. 2018:14(10):577–589. 30171200 10.1038/s41582-018-0058-z

[3] Tukov FF , Mansfield K, Milton M, Meseck E, Penraat K, Chand D, Hartmann A. Single-dose intrathecal dorsal root ganglia toxicity of onasemnogene abeparvovec in cynomolgus monkeys. Hum Gene Ther. 2022:33(13-14):740–756. 35331006 10.1089/hum.2021.255 PMC9347375

[4] Fader KA , Pardo ID, Kovi RC, Somps CJ, Wang HH, Vaidya VS, Ramaiah SK, Sirivelu MP. Circulating neurofilament light chain as a promising biomarker of AAV-induced dorsal root ganglia toxicity in nonclinical toxicology species. Mol Ther Methods Clin Dev. 2022:25:264–277. 35505662 10.1016/j.omtm.2022.03.017 PMC9024379

[5] Sano T , Masuda Y, Yasuno H, Shinozawa T, Watanabe T, Kakehi M. Blood neurofilament light chain as a potential biomarker for central and peripheral nervous toxicity in rats. Toxicol Sci. 2021:185(1):10–18. 34677616 10.1093/toxsci/kfab122 PMC8714368

[6] Meregalli C , Fumagalli G, Alberti P, Canta A, Chiorazzi A, Monza L, Pozzi E, Carozzi VA, Blennow K, Zetterberg H, et al. Neurofilament light chain: a specific serum biomarker of axonal damage severity in rat models of chemotherapy-induced peripheral neurotoxicity. Arch Toxicol. 2020:94(7):2517–2522. 32333051 10.1007/s00204-020-02755-w

[7] Kim SH , Choi MK, Park NY, Hyun JW, Lee MY, Kim HJ, Jung SK, Cha Y. Serum neurofilament light chain levels as a biomarker of neuroaxonal injury and severity of oxaliplatin-induced peripheral neuropathy. Sci Rep. 2020:10(1):7995. 10.1038/s41598-020-64511-5 PMC7224372 32409710

[8] Rodrigues FB , Byrne LM, Tortelli R, Johnson EB, Wijeratne PA, Arridge M, de Vita E, Ghazaleh N, Houghton R, Furby H, et al. Mutant huntingtin and neurofilament light have distinct longitudinal dynamics in Huntington's disease. Sci Transl Med. 2020:12(574):eabc2888. 10.1126/scitranslmed.abc2888 PMC7611886 33328328

[9] Kuhle J , Kropshofer H, Haering DA, Kundu U, Meinert R, Barro C, Dahlke F, Tomic D, Leppert D, Kappos L. Blood neurofilament light chain as a biomarker of MS disease activity and treatment response. Neurology. 2019:92(10):E1007–E1015. 30737333 10.1212/WNL.0000000000007032 PMC6442011

[10] Bacioglu M , Maia LF, Preische O, Schelle J, Apel A, Kaeser SA, Schweighauser M, Eninger T, Lambert M, Pilotto A, et al. Neurofilament light chain in blood and CSF as marker of disease progression in mouse models and in neurodegenerative diseases (vol 91, pg 56, 2016). Neuron. 2016:91(2):494–496. 27477021 10.1016/j.neuron.2016.07.007

[11] Lee S , Yang M, Kim J, Kang S, Kim J, Kim JC, Jung C, Shin T, Kim SH, Moon C. Trimethyltin-induced hippocampal neurodegeneration: a mechanism-based review. Brain Res Bull. 2016:125:187–199. 27450702 10.1016/j.brainresbull.2016.07.010

[12] Lee S , Seo YH, Song JH, Kim WJ, Lee JH, Moon BC, Ang MJ, Kim SH, Moon C, Lee J, et al. Neuroprotective effect of *Protaetia brevitarsis* seulensis' water extract on trimethyltin-induced seizures and hippocampal neurodegeneration. Int J Mol Sci. 2021:22(2):679. 10.3390/ijms22020679 PMC7827571 33445535

[13] Wenger GR , McMillan DE, Chang LW. Behavioral effects of trimethyltin in two strains of mice. II. Multiple fixed ratio, fixed interval. Toxicol Appl Pharmacol. 1984:73(1):89–96. 6710519 10.1016/0041-008x(84)90056-5

[14] Kim J , Yang M, Kim SH, Kim JC, Wang H, Shin T, Moon C. Possible role of the glycogen synthase kinase-3 signaling pathway in trimethyltin-induced hippocampal neurodegeneration in mice. PLoS One. 2013:8(8):e70356. 23940567 10.1371/journal.pone.0070356 PMC3734066

[15] Bolon B , Garman RH, Pardo ID, Jensen K, Sills RC, Roulois A, Radovsky A, Bradley A, Andrews-Jones L, Butt M, et al. STP position paper: recommended practices for sampling and processing the nervous system (brain, spinal cord, nerve, and eye) during nonclinical general toxicity studies. Toxicol Pathol. 2013:41(7):1028–1048. 23475559 10.1177/0192623312474865

[16] Rissin DM , Kan CW, Campbell TG, Howes SC, Fournier DR, Song L, Piech T, Patel PP, Chang L, Rivnak AJ, et al. Single-molecule enzyme-linked immunosorbent assay detects serum proteins at subfemtomolar concentrations. Nat Biotechnol. 2010:28(6):595–599. 20495550 10.1038/nbt.1641 PMC2919230

[17] Ogita K , Nishiyama N, Sugiyama C, Higuchi K, Yoneyama M, Yoneda Y. Regeneration of granule neurons after lesioning of hippocampal dentate gyrus: evaluation using adult mice treated with trimethyltin chloride as a model. J Neurosci Res. 2005:82(5):609–621. 16273549 10.1002/jnr.20678

[18] Sano T , Masuda Y, Yasuno H, Watanabe T, Shinozawa T. Comparative analysis of neurofilament light chain levels in the serum and cerebrospinal fluid in rats subjected to partial sciatic nerve ligation. J Toxicol Pathol. 2023:36(2):145–149. 37101960 10.1293/tox.2022-0110 PMC10123296

